# Is continuous dopaminergic delivery still a major goal to be achieved in the treatment of Parkinson's disease?

**DOI:** 10.1177/1877718X261431104

**Published:** 2026-07-10

**Authors:** Giulia Di Lazzaro, Angelo Tiziano Cimmino, Anna Rita Bentivoglio, Fabrizio Stocchi, Paolo Calabresi

**Affiliations:** 1 UOC Neurology, Fondazione Policlinico Universitario Agostino Gemelli IRCCS, Rome, Italy; 2 Neurology Section, Departments of Neuroscience, Università Cattolica del Sacro Cuore, Rome, Italy; 3 Department of Neurology, University San Raffaele Roma and Institute for Research and Medical Care IRCCS San Raffaele, Rome, Italy

**Keywords:** Parkinson's disease, motor fluctuations, L-DOPA-induced dyskinesias, continuous dopaminergic stimulation, continuous drug delivery

## Abstract

Dopaminergic drugs represent the cornerstone in the symptomatic management of Parkinson's disease motor manifestations, although their long-term use is associated with the appearance of motor fluctuations and dyskinesias. The correlation between pulsatile plasmatic levels of dopaminergic drugs and motor complications suggested that a central continuous dopaminergic stimulation (CDS) could prevent their development. Therefore, several methods have been implemented to provide more stable plasmatic levels of dopaminergic drugs and obtain a continuous drug delivery (CDD). Nevertheless, CDD does not necessarily mean CDS, since other peripheral pharmacokinetic factors intervene before the striatal dopamine release, along with central pharmacodynamic mechanisms. Here, we provide an overview of the different pharmacological approaches taken over time to reach CDS, with limitations and questions that remain open.

## Background

### Historical perspective

Dopaminergic medications have been a cornerstone in managing the symptoms of Parkinson's disease (PD), particularly motor symptoms such as bradykinesia, rigidity, and tremor.^1^ Levodopa (L-DOPA) is a precursor of dopamine (DA) that can cross the blood-brain barrier and replenish dopamine levels in the brain.^2^ In the 1960s, its introduction revolutionized the management of Parkinson's disease. L-DOPA provides significant short-term relief of symptoms, however, in the long term, complications such as motor fluctuations and L-DOPA-induced dyskinesias (LIDs) may appear.^3^

**Figure 1.**
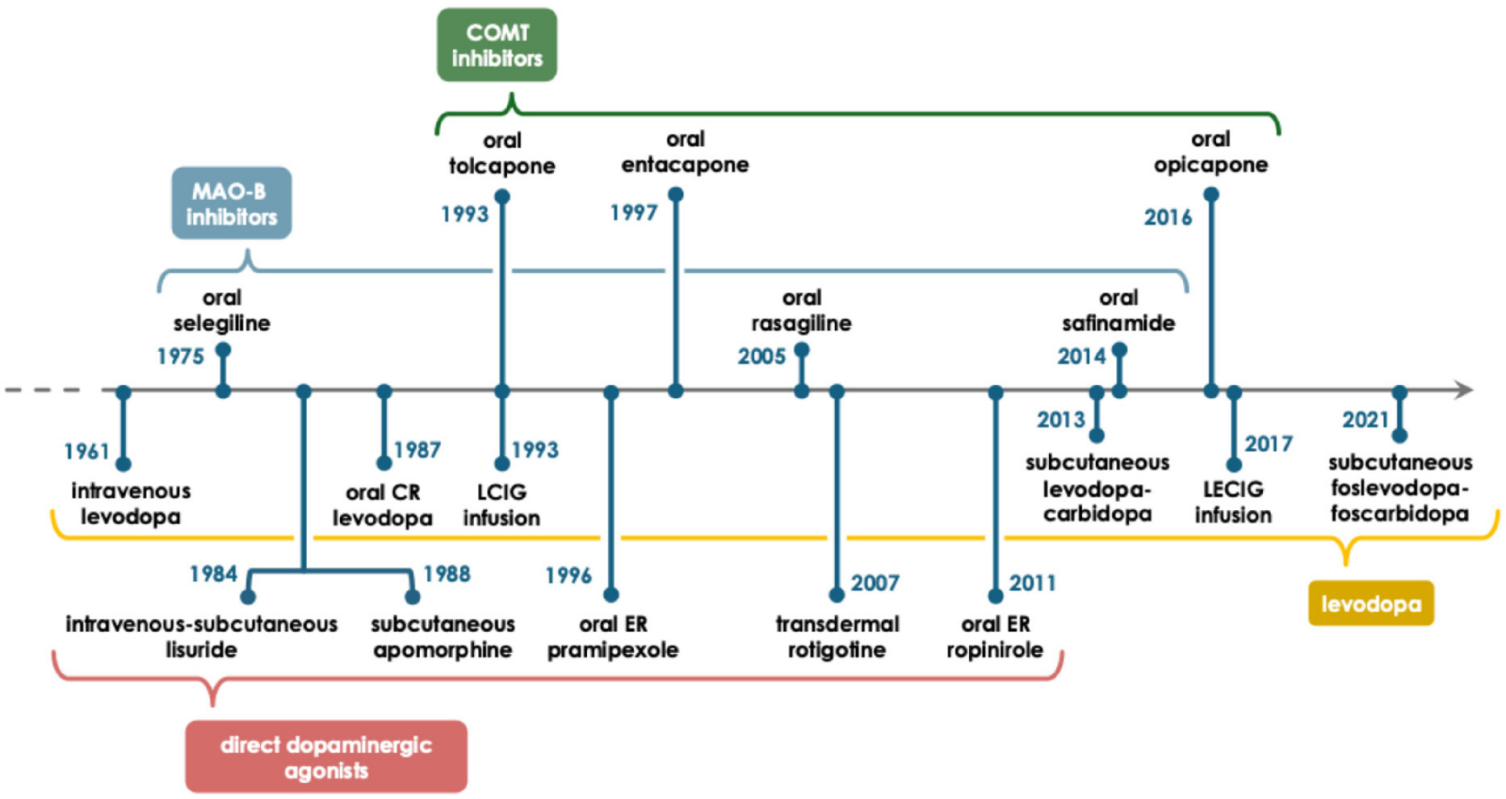
Timeline of the therapeutic strategies implemented for the pursuit of continuous drug delivery (CDD). The four main classes of drugs employed for the symptomatic treatment of PD motor symptoms are represented. Continuous intravenous/subcutaneous administration or extended release (ER) formulations of direct dopaminergic agonists, along with continuous administration strategies of L-DOPA, represent the primary direct methods implemented to achieve stable plasmatic levels of dopaminergic active principles and thus CDD, aiming to achieve continuous dopaminergic stimulation (CDS) and smooth motor fluctuations. Simultaneously, the enzymatic inhibition strategy aims at the achievement of CDS by combining L-DOPA with monoamine oxidase type B inhibitors (MAOB-I), enabling the prolongation of L-DOPA half-life, and/or catechol-O-methyltransferase inhibitors (COMT-I), acting on the inhibition of L-DOPA main peripheral catabolic pathway, ensuring a higher bioavailability of dopamine in the central nervous system. The different drug classes are marked in different colors (COMT-I = green; MAOB-I = blue; L-DOPA formulations = yellow; DA agonists = red). The dates refer to the first published studies on each therapeutic strategy.

It became clear that there was an evident relationship between plasma L-DOPA levels and motor fluctuations.^4^ Therefore, methods to provide more stable plasma levels of L-DOPA were studied to smooth fluctuations (Figure 1). Indeed, continuous intravenous infusion of L-DOPA was studied first.^5^ However, this method was limited by practicality and the risk of peripheral side effects. Then, continuous subcutaneous infusion of the DA agonist lisuride proved to significantly improve fluctuations.^6–8^ The same approach was then used with another potent DA agonist, apomorphine.^9^ The observation that not only L-DOPA but also DA agonists were able to overcome fluctuations suggested that a central continuous dopaminergic stimulation (CDS) was the main factor leading to the improvement of fluctuations and dyskinesias.^6,8,10^ Interestingly, apomorphine was given intravenously in a small group of patients who did not tolerate subcutaneous infusion, with dramatic reduction in OFF time and LIDs, proving that the more constant is the delivery of drug, the better is the control of complications.^11–13^ These experiments in advanced patients were followed by studies with oral DA agonists in early PD versus L-DOPA. All these studies showed that DA agonists in monotherapy induced significantly fewer fluctuations and LIDs. The most accredited explanation for these results was that DA agonists provided a more stable and continuous dopaminergic stimulation compared to oral L-DOPA because of their pharmacokinetic properties.^14–17^ In more recent years, and after years of studies for a jejunal formulation of L-DOPA,^18^ the development of a combination of L-DOPA and carbidopa intestinal gel (LCIG) formulation allowed for continuous delivery directly into the small intestine,^19^ ameliorating motor fluctuations and LIDs in advanced PD. It was also postulated that the reduction of the variability between L-DOPA concentrations independently from the total dose has a role in alleviating fluctuations and LIDs.^20^

Therefore, continuous drug delivery (CDD) and probably consequent CDS can provide significant benefits in managing PD symptoms and motor fluctuations, however, some caveats have been raised over time. Indeed, the mechanisms underlying the development of motor fluctuations and LIDs are not only linked to the pharmacokinetics of oral dopaminergic drugs, since a major determinant of LIDs is the degree of striatal dopaminergic denervation and the consequent aberrant plasticity.^21–24^

Ongoing research continues to explore new methods and technologies for achieving CDS while minimizing side effects and improving patient's outcomes. This includes novel drug delivery systems, gene therapies, and refinements in deep brain stimulation techniques.^25–33^

### The translational role of animal studies

Preclinical studies have demonstrated that CDD can provide sustained dopaminergic stimulation and exert a beneficial effect on motor fluctuations and LIDs as compared to standard oral L-DOPA therapy. Several studies in rodents and MPTP-treated monkeys demonstrated that initial therapy with a long-acting DA agonist was associated with a reduced risk of inducing motor complications than treatment with a short-acting formulation of L-DOPA.^34–36^ It was also demonstrated that continuous exposure to apomorphine could revert the rotational behavior induced by an acute injection of apomorphine in 6-hydroxydopamine (6-OHDA) mice.^37^ However, the symptomatic effect on motor symptoms was higher in animals treated with L-DOPA as compared to DA agonists alone. Therefore, MPTP-treated monkeys were treated with L-DOPA in adjunction to DA agonists; however, it was associated with an increase in the frequency of LIDs in comparison to DA agonist monotherapy, although the frequency remained reduced in comparison to treatment with L-DOPA alone.^38^ Then, CDS through the administration of multiple small doses of L-DOPA plus entacapone was studied in primates.^39^ The authors showed that coadministration with entacapone produced more continuous improvement in locomotor activity with less LIDs than animals treated with L-DOPA four times daily alone, supporting the concept that CDS, even with higher dopaminergic stimulation, could provide beneficial effect on LIDs. However, a more recent study showed that CDD of L-DOPA by intraduodenal infusion in 6-OHDA–lesioned rats can exert a beneficial effect on the expression of established LIDs but did not reduce the risk of LIDs induction.^40^ This evidence supports the concept that different mechanisms could underlie the induction and the expression of motor fluctuations.^41^ Several preclinical studies have suggested that CDS may exert neuroprotective effects in animal models of PD, but the underlying mechanisms remain to be fully elucidated. However, it is hypothesized that sustained dopaminergic stimulation may help stabilize neuronal activity, prevent excitotoxicity, and promote neuronal survival in vulnerable brain regions such as the *substantia nigra pars compacta* (SNpc).^42,43^

Translational studies have also explored the potential of gene therapy approaches for achieving CDS in PD animal models. For instance, viral vector-mediated gene delivery was utilized to overexpress the enzyme aromatic L-amino acid decarboxylase in the striatum of parkinsonian rats,^44^ enhancing DA production and providing sustained motor improvement without the development of LIDs.

Furthermore, optogenetic techniques have been employed in preclinical studies to investigate the effects of precise, temporally controlled stimulation of dopaminergic circuits in animal models of PD. Optogenetic tools were used to selectively activate or inhibit DA neurons in the ventral tegmental area of mice.^45^ This approach elucidated the role of specific neuronal populations in regulating motor behavior and motivated further research into targeted neuromodulation strategies.

These studies highlight the diverse approaches and methodologies used to investigate CDS in models of PD. While further research is needed to validate the neuroprotective effects of CDS in human patients, these preclinical findings provide a rationale for exploring CDS strategies as potential disease-modifying therapies for PD.

## State of the art and future challenges

**Figure 2.**
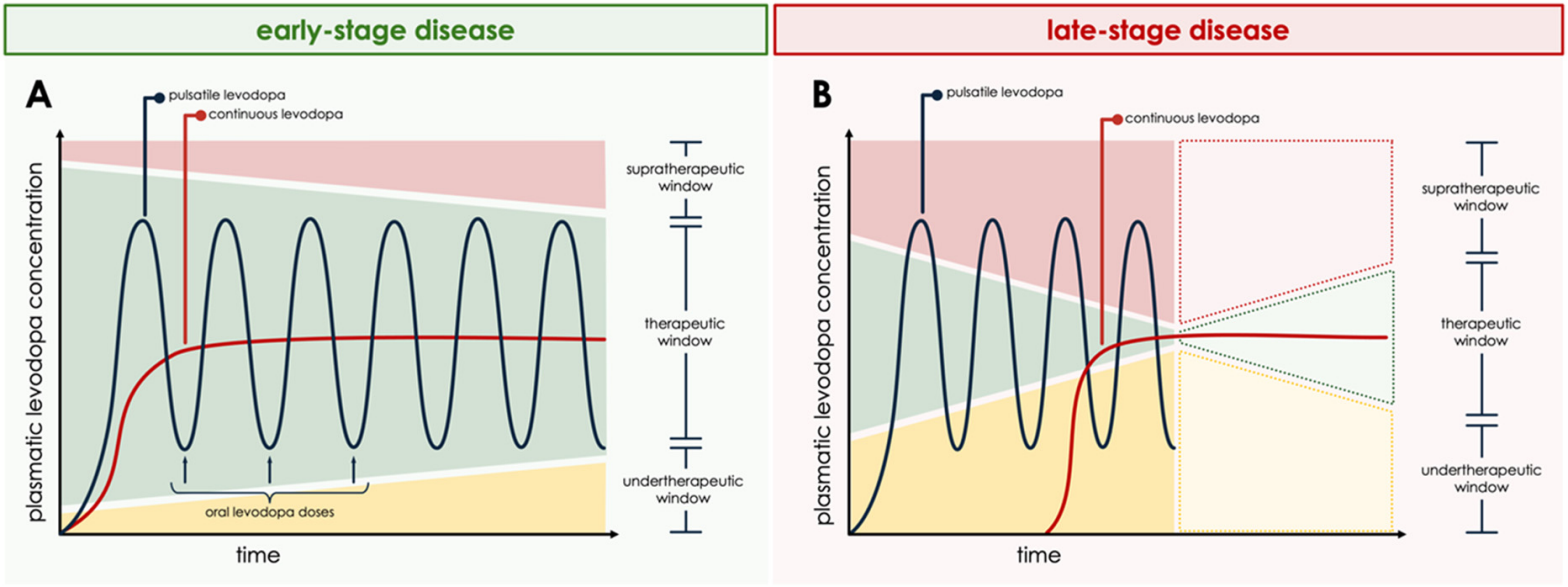
Peripheral plasmatic L-DOPA levels and their relationship with motor fluctuations and dyskinesias. **A**: In early-stage Parkinson's disease (PD), the pulsatile administration of oral L-DOPA at regular intervals is associated with an increase in plasmatic L-DOPA concentrations, followed by subsequent decline until the next dosing. The therapeutic window in this stage is wide, ensuring that fluctuations in plasma L-DOPA concentrations remain within it. Continuous L-DOPA infusion is linked to an increment in plasmatic L-DOPA concentrations until reaching a stable plateau over time, within the therapeutic window. **B:** Throughout PD natural progression, the L-DOPA therapeutic window gradually narrows. Consequently, in late-stage PD, peaks and nadir of plasmatic L-DOPA concentration for each administration fall within supra-therapeutic and under-therapeutic windows, respectively, corresponding to the onset of L-DOPA-induced peak dyskinesia and the wearing-off phenomenon. This process delineates the peripheral mechanism of L-DOPA-induced motor complications. Continuous L-DOPA administration facilitates the maintenance of a stable plasma L-DOPA concentration over time within the therapeutic range, with some evidence supporting the hypothesis of a subsequent reversal of the therapeutic window narrowing (dotted lines).

To date, no disease-modifying treatments are available for PD and L-DOPA remains the most effective symptomatic therapy for motor symptoms in PD. Most patients with PD respond well to oral L-DOPA initially; however, its effectiveness diminishes over time and fluctuations, both motor and non-motor, and dyskinesia appear. As PD progresses, patients begin to alternate periods of good motor control (ON time) and periods of poor motor control and poor mobility (OFF time). Over the years, the use of different dopaminergic drug classes and the design of novel drugs has tried to target the issue of motor fluctuations in PD patients, by giving a more stable dopaminergic stimulation (Figure 2). However, they typically provide only modest benefit for motor fluctuations and therefore this issue remains far from being solved. Several factors contribute to the development of motor fluctuations, being disease duration and the degree of dopaminergic denervation some of the major determinants.^46^

**Figure 3.**
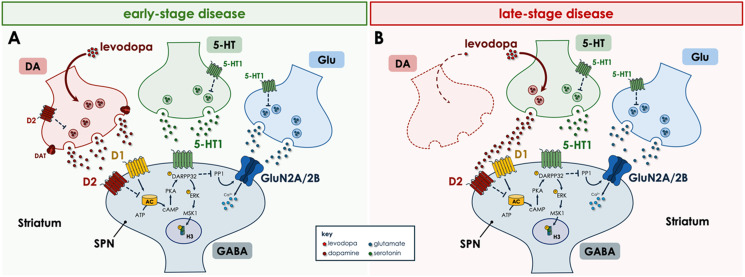
Central pathophysiological mechanisms underlying L-DOPA-induced motor fluctuations and dyskinesias. **A:** In early-stage PD, exogenously administered L-DOPA is uptaken by nigrostriatal dopaminergic neurons and enzymatically converted into dopamine (DA). Subsequently, DA is released into the intersynaptic space, facilitating interaction with D1 and D2 dopaminergic receptors located on the postsynaptic terminals of striatal spiny projection neurons (SPN). D2 autoreceptors on dopaminergic presynaptic terminals control synaptic DA levels through a feedback mechanism. Serotonergic and glutamatergic neurons are not involved in DA release at this stage. **B:** In late-stage PD, the progressive degeneration of dopaminergic neurons leads to the uptake of exogenously administered L-DOPA by serotonergic neurons, where it is converted into DA and released by the presynaptic terminals in an unregulated manner due to the lack of a feedback mechanism mediated by the presynaptic D2 receptors and the activity of the DA transporter (DAT), both absent on the serotonergic neurons. The unregulated D1 dopaminergic receptors hyperstimulation initiates the activation of downstream intracellular molecular cascades, leading to the phosphorylation and activation of DARPP32, which ultimately plays a key role in the regulation of synaptic plasticity and transcriptional activity. Simultaneously, denervated striatal SPNs exhibit an imbalanced ratio of synaptic GluN2A/GluN2B-containing NMDA glutamatergic receptors, with high striatal levels of the GluN2A subunit, potentially contributing to the development of L-DOPA-induced dyskinesia. All these processes represent the main hypothesized central mechanisms of L-DOPA-induced motor complications. 5-HT = serotonin; 5-HT1 = 5-hydroxytryptamine receptor 1; AC = adenylyl cyclase; ATP = adenosine triphosphate; cAMP = cyclic adenosine monophosphate; DAT = dopamine transporter; D1 = dopamine D1 receptor; D2 = dopamine D2 receptor; ERK = extracellular signal-regulated kinase; GABA = γ-aminobutyric acid; Glu = glutamate; GluN2A/2B = N-methyl-D-aspartate receptor subunits 2A and 2B ratio; H3 = histone H3; MSK1 = mitogen- and stress-activated kinase; P-DARPP32 = phosphorylated DA- and cAMP-regulated phosphoprotein 32 kDa; PDE10A = phosphodiesterase 10A; PKA = protein kinase A; PP1 = protein phosphatase 1; SPN = spiny projection neuron.

More recently, CDD has been achieved with infusion pumps of L-DOPA and apomorphine. Despite a more stable plasmatic L-DOPA concentration, patients using those devices continue experiencing some OFF time and LIDs. This could be explained by central mechanisms underlying motor fluctuations and LIDs development, such as aberrant synaptic plasticity,^47^ sensitization of DA receptor subtypes, different expression of ionotropic^48^ and metabotropic^49^ glutamate receptor subunits, as well as other non-dopaminergic neurotransmitter systems^50^ (Figure 3). Moreover, multiple peripheral pharmacokinetic mechanisms can contribute^51^ (Box 1). Taken together, these factors may explain why a CDD could not result in CDS, and therefore not allowing for an optimal PD symptom control.

Box 1. **Peripheral Pathophysiological Mechanisms Underlying Motor Fluctuations and Dyskinesias in PD.**

Motor fluctuations and LIDs in PD are influenced by peripheral pharmacokinetic factors that determine plasma levels of dopaminergic drugs and ultimately the stability of central dopaminergic stimulation. Following oral administration, L-DOPA is absorbed variably in the proximal small intestine via the large neutral amino acid (LNAA) transport system, primarily through the intestinal luminal transporter b^0,+^AT–rBAT (SLC7A9–SLC3A1) and basolateral LAT2-4F2hc (SLC7A8–SLC3A2) and TAT1 (SLC16A10) antiporters. Gastric emptying rate, intestinal motility, and the presence of dietary amino acids competing for these transporters introduce considerable variability in L-DOPA absorption. This variability is further increased due to delayed gastric emptying and slowed intestinal transit, which are typical in PD. Once absorbed, L-DOPA circulates largely free in plasma with negligible protein binding. It is extensively metabolized peripherally via aromatic L-amino acid decarboxylase (AADC) and catechol-O-methyltransferase (COMT), present in the gut wall, liver, and vascular endothelium. This peripheral conversion produces dopamine (which cannot cross the blood–brain barrier) and 3-O-methyldopa (3-OMD), the latter being pharmacologically inactive. Co-administration of AADC inhibitors (e.g., carbidopa or benserazide) and COMT inhibitors reduces peripheral metabolism, prolongs L-DOPA half-life, and increases bioavailability. However, even with enzyme inhibition, fluctuations in plasma concentrations may persist, particularly with intermittent dosing regimens.

L-DOPA is then transported across the blood–brain barrier (BBB) through the same carrier system involved in intestinal uptake: LAT1 (SLC7A5–SLC3A2). Competition with circulating amino acids and plasma level variability directly influence CNS entry. Once inside the brain, L-DOPA is taken up by dopaminergic neurons, converted to dopamine by AADC, and sequestered into synaptic vesicles via VMAT-2, forming the vesicular dopamine pool used for neurotransmission. L-DOPA not transported across the BBB is metabolized peripherally and excreted via renal pathways.

These peripheral dynamics underlie the pulsatile nature of striatal dopaminergic stimulation. As nigrostriatal degeneration progresses, the central buffering capacity diminishes, magnifying the clinical consequences of these fluctuations and predisposing patients to “ON-OFF” phenomena and LIDs. Strategies aimed at CDD are designed to minimize plasma level variability, thereby reducing downstream motor complications.

Here, we provide an overview of the different pharmacological approaches taken over time to provide a more stable dopaminergic stimulation, from the use of DA agonists to the more recent devices for CDD, highlighting the gaps and controversies they have raised. Of note, it is important to highlight that the initial treatment strategies do not necessarily translate into delayed need for device-aided therapies such as DBS, a non-dopaminergic therapy which should be proposed to patients according to well established clinical criteria.^52,53^

### The utility of “direct” dopaminergic agonists

DA agonists can provide CDD through extended-release (ER) oral tablets (pramipexole, ropinirole), transdermal delivery (rotigotine) or subcutaneous infusion (apomorphine). They are also believed to provide a less pulsatile dopaminergic stimulation even in their immediate release (IR) formulation due to their pharmacodynamic properties as compared to oral L-DOPA.

Indeed, several clinical studies have demonstrated the efficacy of ropinirole and pramipexole in improving motor symptoms and reducing motor fluctuations as compared to L-DOPA. With respect to ropinirole, it was demonstrated that, in early PD, it was possible to manage successfully motor symptoms for up to five years with a reduced risk of LIDs by initiating treatment with ropinirole alone and supplementing it with L-DOPA if necessary.^16^ However, once the L-DOPA was started, the authors could not demonstrate a persisting preventive effect.^54^ It was later shown that ropinirole ER was superior to ropinirole IR in controlling motor fluctuations as adjunctive therapy to low doses of L-DOPA.^55^ With regard to pramipexole, the CALM-PD trial^14^ demonstrated that it reduced the risk of motor fluctuations compared to L-DOPA in early PD patients. Then, the pramipexole ER formulation^56^ was proven not to be inferior to the IR formulation.

Rotigotine is a non-ergolinic DA agonist that provides CDD through transdermal delivery. A randomized, double-blind, placebo-controlled trial^57^ demonstrated that rotigotine significantly reduced OFF time and improved motor function compared to placebo in early PD patients, suggesting its potential role in mitigating motor fluctuations. A long-term follow-up study^58^ showed sustained improvements in motor function and reductions in OFF time in advanced PD patients.

D1/D5 dopamine receptor agonists are pharmacological agents that selectively activate the D1-like family of dopamine receptors, which includes D1 and D5 subtypes.^59,60^ These receptors are Gs-coupled and primarily stimulate adenylyl cyclase, increasing intracellular cAMP, and are implicated in motor control, cognition, and neuropsychiatric function. D1/D5 agonists are under investigation for Parkinson's disease, cognitive impairment in schizophrenia, and other CNS disorders, but none are yet approved for routine clinical use. Tavapadon, a novel oral dopamine-D1R/D5R partial agonist, has been studied in recent years for the treatment of late-stage development PD. In placebo-controlled trials, Tavapadon produced clear, clinically meaningful gains in motor function and day-to-day activities, as captured by the Unified Parkinson's Disease Rating Scale (UPDRS).^61,62^ Recent late-stage results have revealed that Tavapadon maintains superior UPDRS outcomes in de novo patients and, when added to levodopa, extended “ON” time periods of reliable motor control free of troublesome dyskinesia. Currently, phase 3 trials, such as the TEMPO-4 study, are evaluating its long-term efficacy in PD patients. This study is currently enrolling by invitation and is expected to be completed in January 2026.^63^

Overall, clinical evidence suggests that ER DA agonists therapy may be effective in reducing motor fluctuations and improving motor function in patients with PD. However, especially in the advanced stages of the disease, a DA agonist monotherapy is not able to give a good symptom control, and a combination with oral L-DOPA is needed, with a consequent increase of the incidence of motor fluctuations. Moreover, the use of DA is also limited by the non-motor complications, such as impulse-control disorder, daytime sleepiness and nausea.^64^ Indeed, the role in alleviating motor fluctuations is mostly attributable to the dopaminergic effect than to a specific anti-dyskinetic pharmacodynamic mechanism.

### The lesson from MAO-B inhibitors

Monoamine oxidase type B (MAO-B) inhibitors, such as selegiline, rasagiline and safinamide, work by inhibiting the enzyme MAO-B, responsible for the degradation of DA and other monoamines in the central nervous system. Therefore, MAO-B inhibitors have been studied for their potential effects on motor fluctuations. Overall, both selegiline and rasagiline are associated with reductions in OFF time and improvements in motor function compared to placebo or other antiparkinsonian medication.^65–67^ Safinamide is a MAO-B inhibitor approved for the use as adjunct therapy to levodopa/carbidopa in PD patients. It also modulates glutamate release, potentially providing benefits in managing motor fluctuations in PD patients. Two Phase III clinical trials demonstrated the efficacy and safety of safinamide as add-on therapy to L-DOPA in patients with mid- to late-stage PD experiencing motor fluctuations.^68,69^ A post-hoc analysis examined the effects of safinamide on motor fluctuations and LIDs in PD patients,^70^ showing an improvement in LIDs. The authors postulated that the results could be related to safinamide inhibition of sodium channels and stimulated glutamate release, therefore widening the perspective also to other possible mechanisms underlying motor fluctuations and LIDs rather that CDS.

### The lesson from COMT inhibitors

In 1997, a study on tolcapone use in PD patients with motor fluctuations showed a significant reduction in OFF time and improvements in motor function as compared to placebo.^71^ Few years later, also entacapone was demonstrated to significantly reduce OFF time and increase ON time without troublesome dyskinesias when added to L-DOPA therapy.^72,73^ In 2016, opicapone was approved for medical use in the European Union. Two randomized, double-blind, placebo-controlled trials^74,75^ demonstrated its efficacy and safety in patients with PD experiencing motor fluctuations. More recent evidence is supporting its beneficial effect also in earlier phases of the disease, when only minor fluctuations are present.^76,77^

Catechol-O-methyltransferase (COMT) inhibitors (COMT-I) work by inhibiting the enzyme COMT, the main peripheric catabolic pathway for L-DOPA.^78^ Thanks to their pharmacodynamic properties, they are thought to provide a less pulsatile dopaminergic stimulation. However, the failure of the STRIDE PD study^79^ is a good example of the complexity of the challenge to reduce the incidence of motor fluctuations and LIDs by providing a more stable dopaminergic stimulation. In this trial, the authors performed a prospective 134-week double-blind study comparing the risk of developing LIDs in PD patients randomized to initiate L-DOPA therapy with L-DOPA/carbidopa or L-DOPA/carbidopa/entacapone, administered 4 times daily at 3.5-h intervals. At the end of the study, the group taking the COMT-I developed LIDs significantly earlier and with shorter interval. However, the authors argued that the trial design was probably not adequate. One issue was that the patients taking the COMT-I were exposed to higher L-DOPA-equivalent daily dose, a well-acknowledged risk factor for LIDs development, and the fact that a 3.5-h interval was not short enough to prevent dopaminergic pulsatile stimulation.^79^

### Learning from extended release L-DOPA formulations

In the United States, extended-release oral L-DOPA formulations such as Rytary^®^ (IPX066) and the more recently approved Crexont^®^ (IPX203) have been developed to prolong L-DOPA plasma exposure and reduce OFF time.^80–82^ Rytary^®^ combines immediate-release and extended-release beads within a single capsule, resulting in a faster onset of action together with a more sustained L-DOPA concentration compared with immediate-release formulations.^83^ Crexont^®^ was specifically designed to further extend L-DOPA duration of action and allow longer dosing intervals.^84^ Clinical studies have shown that both formulations can reduce OFF time and dosing frequency and improve motor symptom control. However, despite smoother peripheral pharmacokinetics, these oral extended-release formulations still rely on gastrointestinal absorption and intermittent administration and therefore do not achieve true CDD or CDS.

### Learning from apomorphine and LCIG infusions

Continuous subcutaneous apomorphine infusion (CSAI) is an advanced treatment option primarily used in the management of motor fluctuations and LIDs associated with advanced PD. CSAI involves the placement of a small subcutaneous infusion pump, which delivers a continuous infusion of apomorphine throughout the day. Since it is administered subcutaneously, it bypasses the gastrointestinal tract, making it suitable for patients with absorption issues or severe nausea related to oral medications. Close monitoring by healthcare providers is essential to optimize therapy and minimize adverse effects, such as nausea, hypotension, and skin reactions at the infusion site. CSAI was the first infusion therapy available in Europe and its introduction to the market dates back to the early 1990s.^9,85^ In 2001, the efficacy and safety of CSAI in PD patients with motor fluctuations was evaluated.^86^ The study demonstrated that CSAI significantly reduced OFF time and improved motor function compared to placebo. A long-term follow-up study^13^ assessed the durability of CSAI over 12 months, showing a sustained improvement in motor fluctuations and quality of life with continued apomorphine treatment. However, the first large randomized, placebo-controlled study, the TOLEDO trial,^87^ came only years later. It found that patients receiving CSAI compared to placebo showed a 1.9-h reduction in OFF time with no worsening in troublesome LIDs, and the prolongation of ON time without troublesome LIDs. Unfortunately, no significant benefit on quality of life was observed. Indeed, the incidence of any treatment-emergent adverse event by week 12 was 93% in the apomorphine subcutaneous infusion group versus 57% in the placebo group. Infusion-site reactions (including erythema, nodules, and cellulitis) were the most common adverse events, occurring in 35% of patients on apomorphine versus 7% on placebo. Neuropsychiatric adverse events (including confusion, hallucinations, psychosis, hypersexuality, and punding) occurred in 15% of patients on apomorphine versus 9% on placebo. Serious adverse events occurred in 9% of the apomorphine group and 4% of the placebo group. Adverse events leading to withdrawal occurred in 11% of the apomorphine group and 0% of the placebo group. Considering the non-motor aspects, CSAI may also show a beneficial effect on mood/apathy, perceptual problems, memory and sleep.^88^ However, these results are not always paralleled by a better quality of life of advanced PD patients.

LCIG infusion therapy is another treatment option for advanced PD patients experiencing motor fluctuations and LIDs. It involves delivering a continuous infusion of L-DOPA/carbidopa gel directly into the small intestine via a portable pump system. In 2014, the efficacy and safety of LCIG in advanced PD patients with motor fluctuations was investigated in a randomized controlled trial, demonstrating a significant reduction in the OFF time and improvement in motor function compared to standard oral L-DOPA therapy.^19^ A long-term extension of the trial^89^ evaluated the durability of LCIG therapy over 54 weeks, showing a sustained improvement in motor fluctuations and quality of life. LCIG has also a clear effect at the level of non-motor symptoms and particularly for sleep, mood, and gastrointestinal problems, and on overall quality of life of both patients and caregivers.^90,91^ However, LCIG requires a surgical procedure to implant the PEG-J tube and carry the risk of device-related complications, such as tube dislocation, infection, or occlusion.^90^ Moreover, an increased risk for the development of or deterioration of pre-existing polyneuropathy has been reported.^92^

### The hope of subcutaneous L-DOPA infusions

As extensively discussed above, despite the existence of different CDD approaches, a significant unmet need exists for patients with advanced PD to have an individualized, continuous and non-surgical therapy that provides symptomatic relief via the predictable delivery of L-DOPA.

In this context, in 2021, the first phase I studies on safety and tolerability of foslevodopa/foscarbidopa (also referred to as LDp/CDp or ABBV951) were published.^93,94^ After subcutaneous infusion, a stable L-DOPA pharmacokinetic profile was maintained, and the infusion was well tolerated.^93,94^ A phase III trial studied the efficacy of LDp/CDp delivered as a 24-h/day continuous subcutaneous infusion (CSCI) for the treatment of motor fluctuations in PD.^95^ LDp/CDp CSCI provided a significant increase in ON time without troublesome LIDs and decrease in OFF time at week 12 compared with oral L-DOPA. However, no improvement was found in the MDS-UPDRS III scores. Most of the reported adverse events were related to the infusion site (erythema, pain, cellulitis and oedema). Also, hallucinations or psychosis were reported more frequently in the LDp/CDp group.

Another drug-device combination, not yet commercially available, consisting of a sterile solution of L-DOPA/carbidopa continuously delivered via a dedicated subcutaneous pump is ND0612. Feasibility, safety and preliminary evidence of the efficacy of ND0612 infusion for 24-h and 14-h daily come from a 28-day open-label study (NCT0257752).^96^ Another study with the primary aim of evaluating the long-term safety and tolerability of ND0612 is the BeyoND study (NCT02726386).^97^ Its data indicate an overall good safety profile, but infusion site reactions related to the subcutaneous route of administration are common and may lead to treatment discontinuation. Recently, results from the BouNDless study, a phase III randomized, active-controlled, double-blind, double-dummy trial were released, showing that treatment with ND0612 was superior over oral L-DOPA/carbidopa, with higher “Good ON” time.^98^

Overall, CSCI of L-DOPA formulations has several strengths. First, being a subcutaneous infusion, it allows to avoid the limits related to gastrointestinal absorption of the drugs. Several PD patients may experience gastrointestinal absorption issues, which can affect the effectiveness of oral medications, including L-DOPA. Subcutaneous infusion bypasses the gastrointestinal tract, ensuring consistent drug delivery and avoiding problems related to erratic absorption (Box 1). For patients who have difficulty swallowing, subcutaneous and jejunal infusions offer an alternative route of administration, reducing the oral medication burden. Safety, also in advanced patients, is acceptable.^99–101^ Finally, a 24-h infusion provides a more stable and prolonged duration of the dopaminergic effect, helping to maintain consistent symptom control throughout the day and the night.

## Conclusions

CDD is one of the major strategies employed to manage motor fluctuations and LIDs, complications that develop virtually in every patient with PD over time. The early initiation of CDD represents a biologically plausible approach to minimize pulsatile dopaminergic stimulation. However, available clinical evidence does not demonstrate a clear preventive effect on the development of motor fluctuations, suggesting that disease progression and central maladaptive plasticity outweigh peripheral pharmacokinetic stabilization. Notwithstanding, to date, CDD represents one of the best strategies to address motor fluctuation, from a symptomatic point of view. Several strategies have been employed to pursue CDD, from extended-release oral dopaminergic drugs to enzymatic inhibitors, which allow a longer half-life or higher bioavailability of DA in the CNS, to jejunal or subcutaneous infusion of dopaminergic drugs (Figure 1). Among the latter, CSCI of L-DOPA has some potential advantages since it allows for a 24-h infusion, easier to administer and bypassing the gastrointestinal tract. Other delivery systems are under investigation, such as the intrabuccal continuous delivery system,^102^ and innovative drug delivery systems, such as polymeric microparticles and nanoparticles that allow for the extended-release of the active principles targeting the desired site of actions,^103–110^ both still at an investigational stage. However, all these approaches have taken into account only some of the mechanisms underlying the emergence of motor fluctuations, mainly the peripheral mechanisms (e.g., erratic intestinal absorption of dopaminergic drugs, fluctuating plasmatic levels) (Box 1). In this regard, the CDD could not be mirrored by a CDS, since many pharmacokinetic factors, such as biodistribution and enzymatic conversion, intervene before the DA is released by the presynaptic terminal. Moreover, central mechanisms such as aberrant striatal plasticity, neuroinflammation, DA receptor subtype imbalance, abnormal ionotropic and metabotropic glutamate receptors subunit expression, along with the contribution of other non-dopaminergic neurotransmitter systems, should be targeted (Box 2).

Box 2. **Central Pathophysiological Mechanisms Underlying Motor Fluctuations and Dyskinesias in PD.**

The degeneration of nigrostriatal dopaminergic neurons in PD leads to a progressive loss of the physiological storage and regulated release of DA. As this presynaptic buffering capacity declines, exogenous L-DOPA is increasingly taken up and converted to DA by non-dopaminergic neurons, particularly serotonergic terminals. These neurons lack presynaptic D2 autoreceptors and dopamine transporters (DAT), preventing feedback regulation and reuptake. Thus, DA is released in a pulsatile and unregulated manner, contributing to the erratic stimulation of post-synaptic DA receptors and, therefore, to the appearance of motor fluctuations and LIDs.

At the postsynaptic level, intermittent and unregulated dopaminergic stimulation induces profound maladaptive changes in striatal transmission. Overactivation of D1 receptors in spiny projection neurons (SPNs) results in the activation of cAMP–PKA pathway, phosphorylation of DARPP-32, and sustained ERK signaling. ERK, via MSK1 and histone H3 phosphorylation, ultimately induces chromatin remodeling and facilitates the transcription of genes that promote long-lasting alterations in synaptic function and structure. This same signaling cascade leads to a peculiar shift in NMDA receptor subunit composition, with an increased GluN2A-to-GluN2B ratio at corticostriatal synapses, modifying calcium influx kinetics and lowering the induction threshold for LTP. In parallel, abnormal stimulation of group I metabotropic glutamate receptors (mGluR1/5) activates PLCβ pathway, ultimately leading to the dysregulation of endocannabinoid-mediated retrograde signaling. As a consequence of these post-synaptic changes, the physiological synaptic plasticity is disrupted: LTD is lost or markedly reduced, while LTP becomes abnormally stabilized. Structurally, this manifests as dendritic spine remodeling on spiny SPNs.

The complex interplay between unregulated presynaptic DA release, postsynaptic transcriptional reprogramming, NMDA receptor subunit imbalance, and mGluR-driven signaling abnormalities forms the core of the central mechanisms underlying motor fluctuations and dyskinesias in PD. CDD approaches aim to provide a more physiological dopaminergic tone, which may limit aberrant serotonergic release, normalize glutamatergic drive, and restore physiological synaptic plasticity.

Is then CDS still a major goal to be achieved in the treatment of PD? Yes, but if advances have been made in CDD, CDS is still far from being reached. Figures 2 and 3 show the difference between CDD and CDS. The peripheral level of drug used to stimulate central DA receptors directly or indirectly may be improved by CDD, reducing motor fluctuations as well as LIDs. It has been even postulated that the shift from pulsatile blood levels of drugs acting on PD symptoms to stable peripheral levels of dopaminergic drugs may revert the narrowing of the therapeutic window^111^ (Figure 2). Nevertheless, we are aware that CDD does not mean CDS. In fact, central pathophysiological mechanisms deeply influence the effect of dopaminergic drugs. Firstly, sensitization of D1 receptors and downstream intracellular mechanisms are implicated in motor fluctuations and LIDs. Secondly, after dopaminergic striatal terminal loss, the aberrant sprouting of serotonergic terminals causes the release of endogenous DA in an uncontrolled manner; these terminals, in fact, are unable to buffer the abnormal release of DA because they lack the DA transporter, since this regulatory mechanism is only present in dopaminergic terminals.^23,112–115^ Finally, the denervated striatum, after chronic L-DOPA treatment, exhibits a form of aberrant synaptic plasticity such as the lack of reversal plasticity (lack of depotentiation), which is considered a major problem for erasing unessential motor information^22,116^ (Figure 3).

To date, CDD remains the best option to tackle the emergence of motor fluctuations and LIDs in PD patients. Approaches such as the combination of different pharmacological therapies, both dopaminergic and non-dopaminergic such as DBS, and non-pharmacological interventions, such as lifestyle changes^117^ may be able to better address central and peripheral mechanism underlying motor fluctuations, but consensus is still lacking. Further studies on novel drugs and non-pharmacological approaches, which could address more specifically the known central pathogenic mechanisms, are still needed.
